# Supplementary figures and images for: Variations in microbiota composition of laboratory mice influence Citrobacter rodentium infection via variable short-chain fatty acid production
Source: PLoS Pathog. 2020 Mar 24;16(3):e1008448. doi: 10.1371/journal.ppat.1008448 (PMC7141690; doi:10.1371/journal.ppat.1008448)

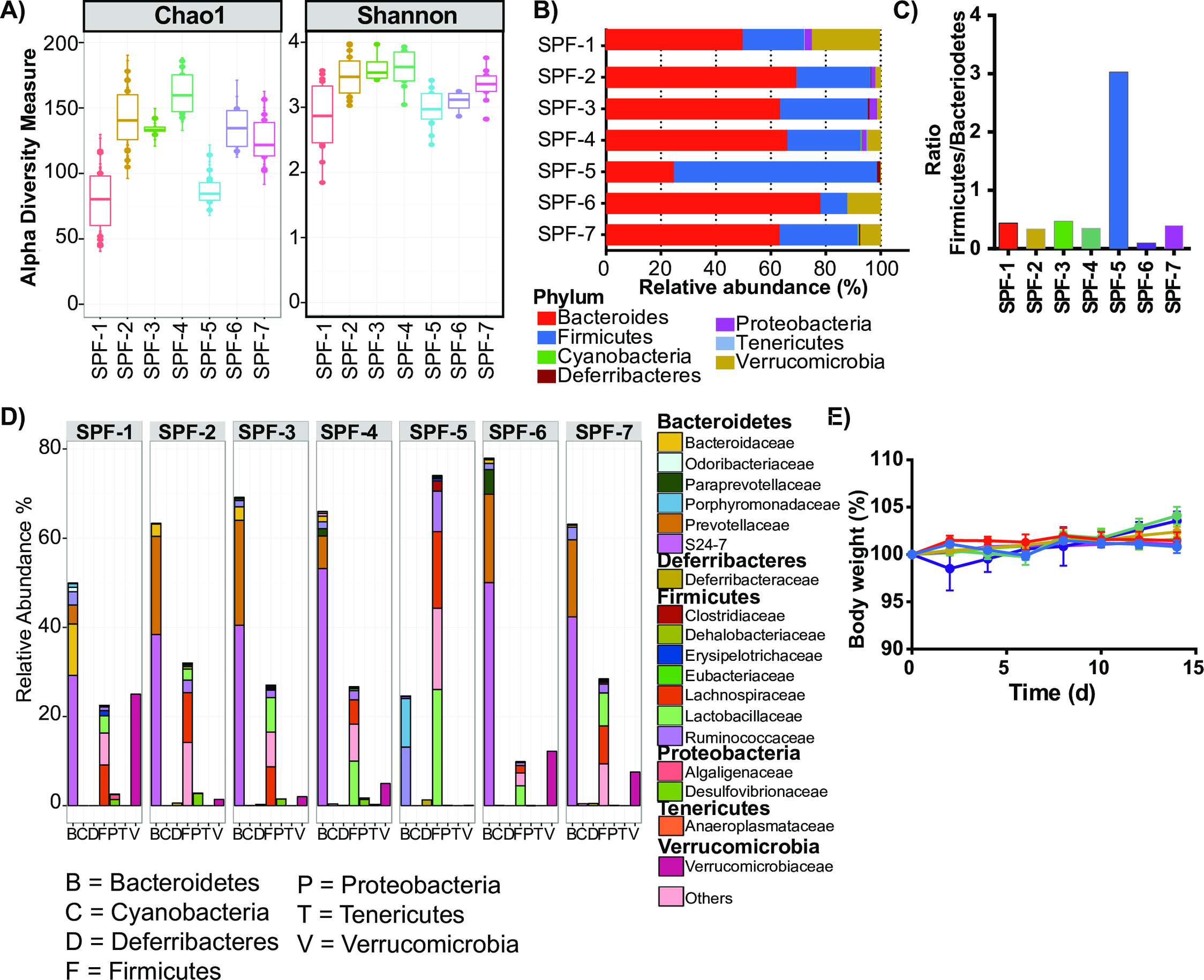

Supplement: S1 Fig — Fecal microbiota of different specific pathogen free (SPF) mouse lines including SPF-1/SPF-S (n = 17), SPF-2/SPF-R (n = 18), SPF-3 (n = 7), SPF-4 (n = 9), SPF-5 (n = 15), SPF-6 (n = 4) and SPF-7 (n = 12) were evaluated using 16S rRNA gene sequencing. (A) α-diversity was determined using Chao1 and Shannon index. (B) Relative abundance at phylum level for each group is shown. (C) Ratio of taxonomic order Firmicutes and Bacteroidetes for each group. Representative data derived from one experiment (SPF-4, SPF-6) or are pooled from at least two different experiments (SPF-1, SPF-2, SPF-3, SPF-5, SPF-7). P values indicated represent a nonparametric Kruskal-Wallis test with multiple comparisons (one-way ANOVA). *p<0.05, **p<0.01, ***p<0.001, ****p<0.0001. (D) Relative abundances of bacterial families are shown and grouped according to their phylum. Bars represent the mean of all mice within the group. Representative data derived from one experiment (SPF-4, SPF-6) or are pooled from at least two different experiments (SPF-1, SPF-2, SPF-3, SPF-5, SPF-7). (E) Mice with different microbiota settings (SPF-1-SPF-7) were infected orally with 108 CFU C. rodentium. Body weight was recorded during the course of infection. Results represent n = 4–18 mice/group as mean ± SEM from one experiment (SPF-4, SPF-6) or pooled from at least two different experiments (SPF-1, SPF-2, SPF-3, SPF-5, SPF-7). (TIF) [file ppat.1008448.s001.tif]

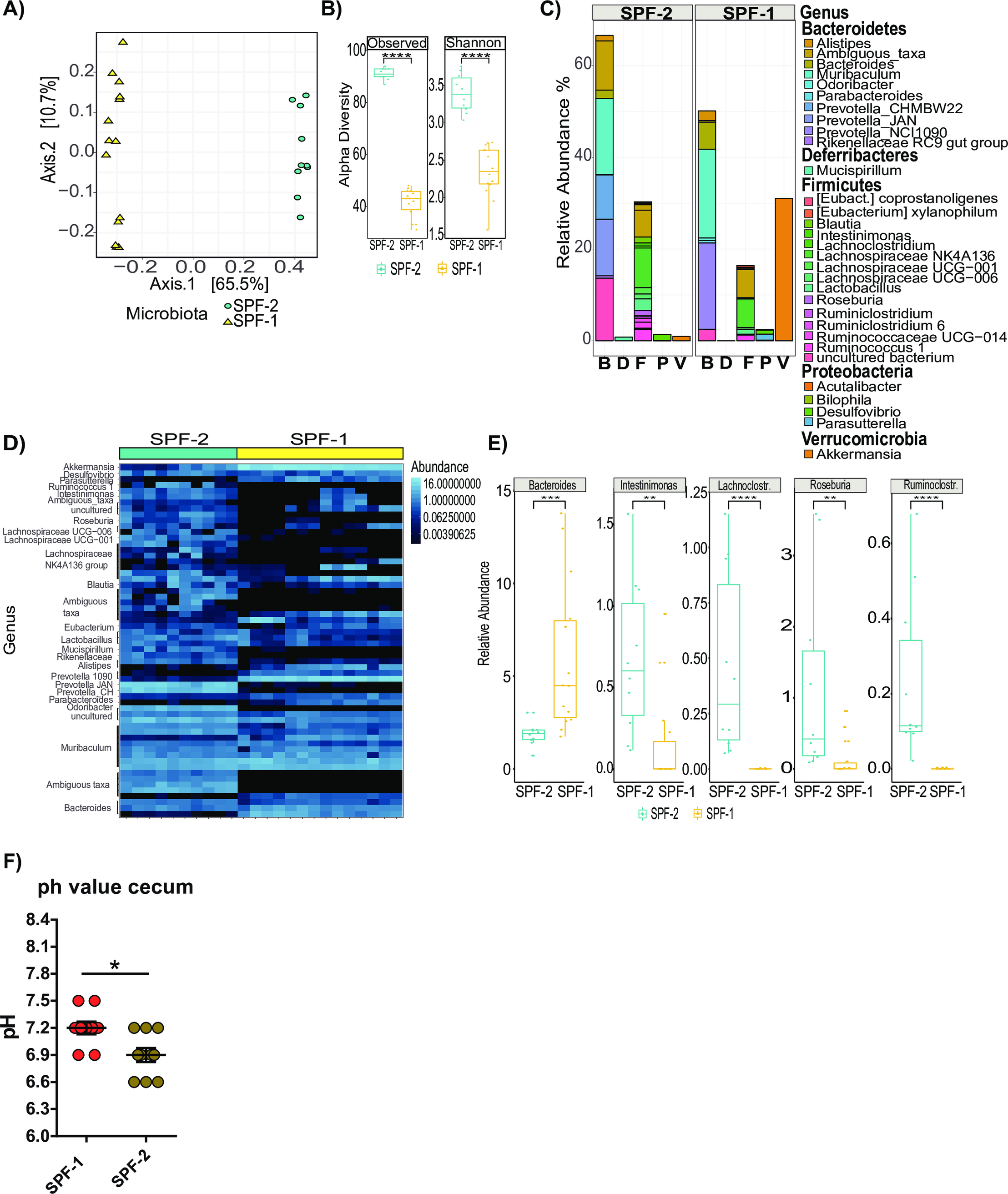

Supplement: S2 Fig — (A) Fecal microbiota of resistant SPF-R and susceptible SPF-S mice was analyzed using 16S rRNA gene sequencing using a principal coordinates analysis (PCoA) plot (B) α-diversity was determined using Chao1 and Shannon index. P values indicated represent a non-parametric Wilcoxon signed rank test ****p<0.0001. (C) Relative abundances are displayed at genus level. (D) Heatmap of relative abundances of bacterial genus (≥ 97% sequence similarity, > 0.05% relative abundance) is sorted by genus and mouse line. Each vertical bar represents the microbiota of an individual mouse. Representative data are from one experiment out of three independent experiment. (E) Relative abundance of significantly different SCFA producing members between SPF-S and SPF-R of the genus Bacteroides, Intestinimonas, Lachnoclostridium, Roseburia and Ruminoclostridium are shown. P values indicated represent a non-parametric Wilcoxon signed rank test *p<0.05, **p<0.01, ***p<0.001, ****p<0.0001. (F) pH value of the cecum at steady state in susceptible SPF-S and resistant SPF-R mice. P values indicated represent a Mann-Whitney U test comparison between groups with *p<0.05. (TIF) [file ppat.1008448.s002.tif]

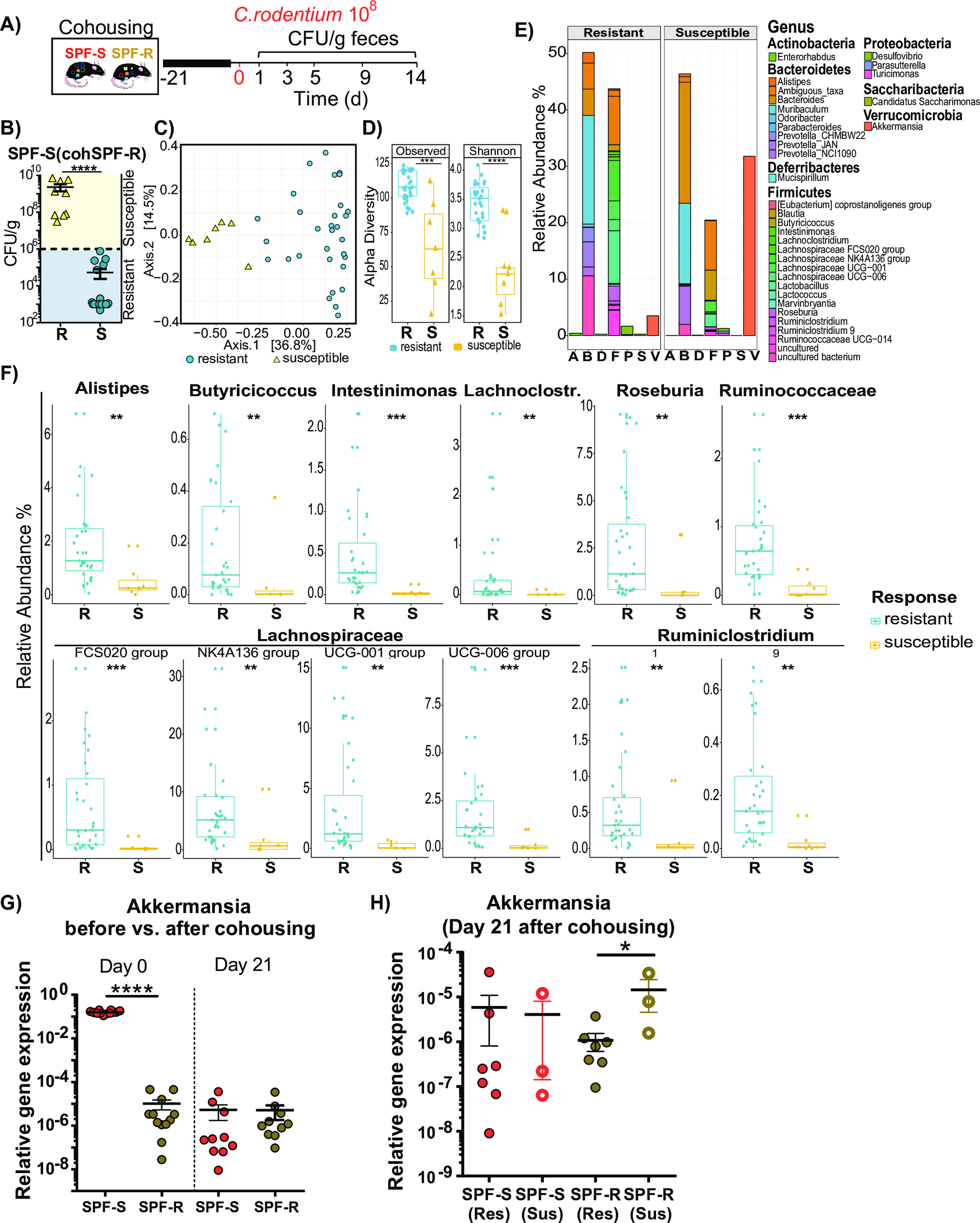

Supplement: S3 Fig — (A) SPF-S and SPF-R mice were cohoused for 4 weeks and infected with 108 CFU C. rodentium. (B) Assignment of cohoused SPF-S and SPF-R mice to the class “resistant” and “susceptible” according to the cecal CFUs of C. rodentium after day 3 p.i.. A threshold of 106 was used for discrimination of both groups. (C) Fecal microbiota was analyzed using 16S rRNA gene sequencing after cohousing using a principal coordinates analysis (PCoA) plot. (D) α-diversity was determined using Chao1 and Shannon index. (E) Fecal microbiota of resistant and susceptible cohoused SPF-S and SPF-R mice was analyzed using 16S rRNA gene sequencing. Relative abundances of bacterial families are shown and grouped according to their phylum. Bars represent the mean of all mice within the group. Representative data derived from three independent experiment are pooled. (F) Relative abundance of significantly different SCFA producing members between resistant and susceptible mice of the genus Alistipes, Butyricicoccus, Intestinimonas, Lachnoclostridium, Lachnospiraceae, Roseburia, Ruminococcaceae and Ruminoclostridium are shown. P values indicated represent a non-parametric Wilcoxon signed rank test *p<0.05, **p<0.01, ***p<0.001, ****p<0.0001. (G) Relative levels of Akkermansia in SPF-S and SPF-R animals before and after three weeks of cohousing. Values are normalized to total 16S. (H) Relative levels of Akkermansia cohoused SPF-S and SPF-R mice that changed or maintained the initial phenotype. Values are normalized to total 16S. P values indicated represent a non-parametric Wilcoxon signed rank test *p<0.05 (TIF) [file ppat.1008448.s003.tif]

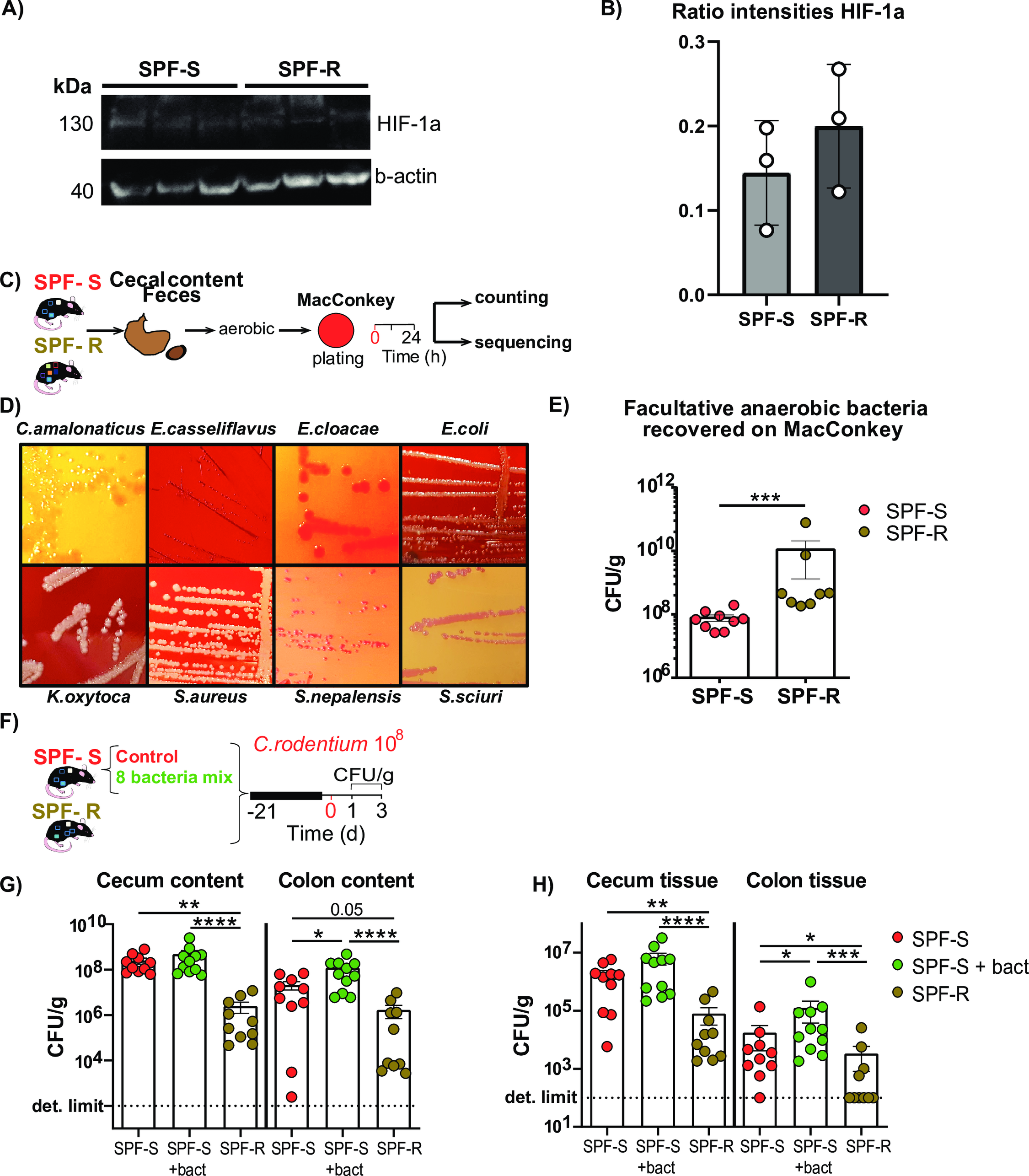

Supplement: S4 Fig — (A) HIF-1 α protein from cecum tissue at steady state in SPF-R and SPF-S mice (B) Quantification of HIF-1 α protein compared to β-actin protein levels (loading control) (C) Serial dilutions of isolated cecal content and feces of SPF-S and SPF-R mice were plated on MacConkey agar plates without crystal violet and cultivated for 24 hours under aerobic conditions. Morphological different colonies were picked and streaked out again to obtain a pure culture. Single colonies were sent for sequencing. All aerobic colonies were counted for quantification (D) Isolated bacteria grown on Mac Conkey agar plates. (E) Quantification of cultivable aerobic bacteria in the feces of SPF-1 and SPF-2 mice growing on Mac Conkey agar plates media. Data represent two independent experiments with n = 5–8 mice per group. (F) Groups of susceptible SPF-S mice were pretreated with isolated facultative anaerobic bacteria. All mice were infected with 108 CFU C. rodentium after 3 weeks of precolonization and CFUs/g organ content and tissue were assessed after 3 days p.i. (G-H) CFUs of C. rodentium in intestinal organ tissues and contents after 3 days p.i.. Results represent Mean and SEM of two independent experiments with n = 6–10 mice per group. P values indicated represent a nonparametric Kruskal-Wallis test *p<0.05, **p<0.01, ***p<0.001, ****p<0.0001. (TIF) [file ppat.1008448.s004.tif]

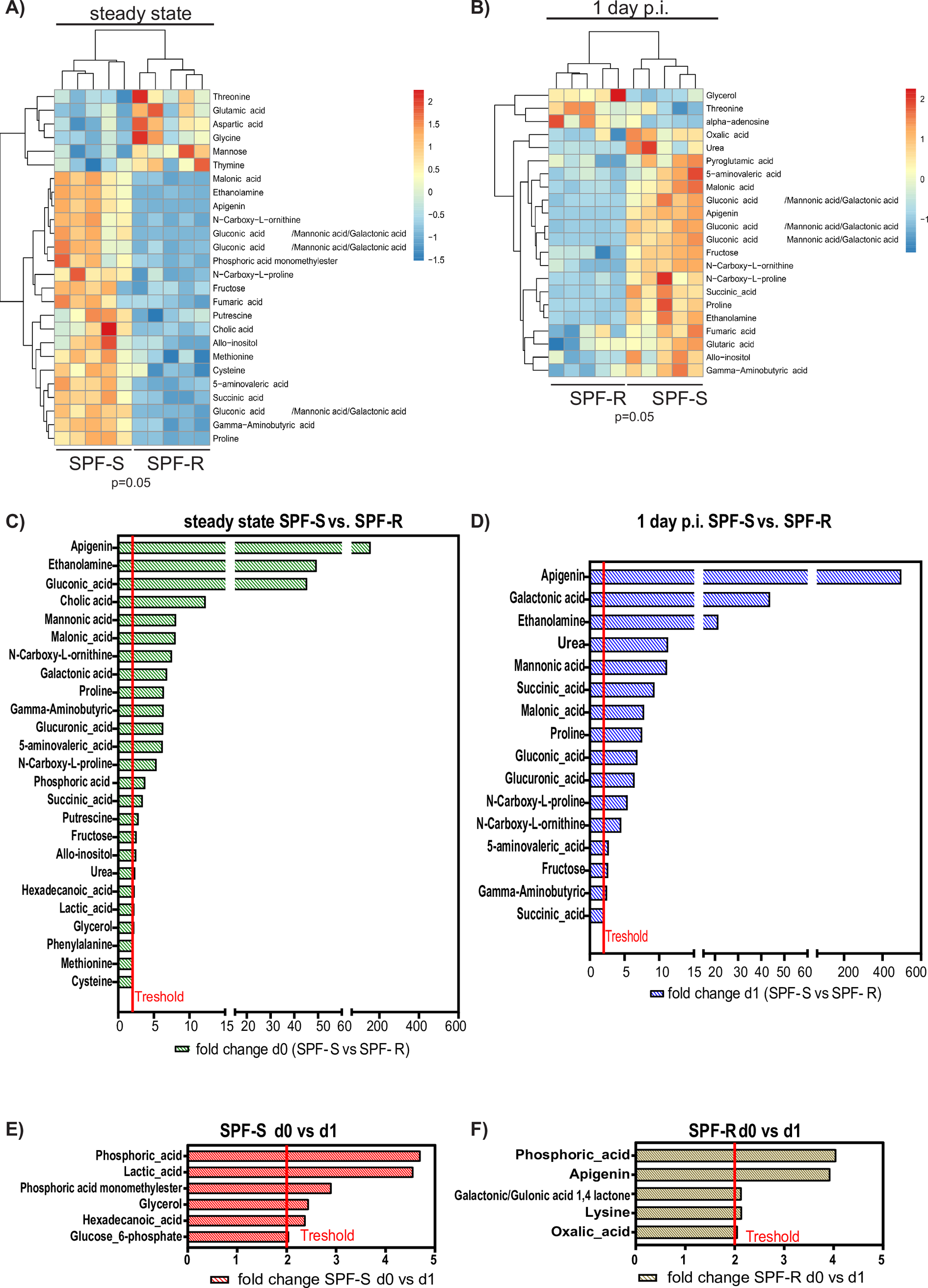

Supplement: S5 Fig — Heat maps of successfully annotated and significantly different metabolites between SPF-S and SPF-R mice at different time points: steady state (A) and day 1 p.i. (B). The two dendrograms for the heat map were calculated using Euclidean distance and ward linkage. t-tests with *p<0.05 were performed pairwise and significantly different metabolites between two extraction solvents are indicated. Fold changes of successfully annotated and significantly different metabolites between SPF-S and SPF-R at steady state (C) or at day 1 p.i. (D) are displayed. Fold changes of successfully annotated and significantly different metabolites within a mouse lines at different time points: SPF-S (E) and SPF-R (F). More than 2-fold changes between the indicated groups were set as a threshold. (TIF) [file ppat.1008448.s005.tif]

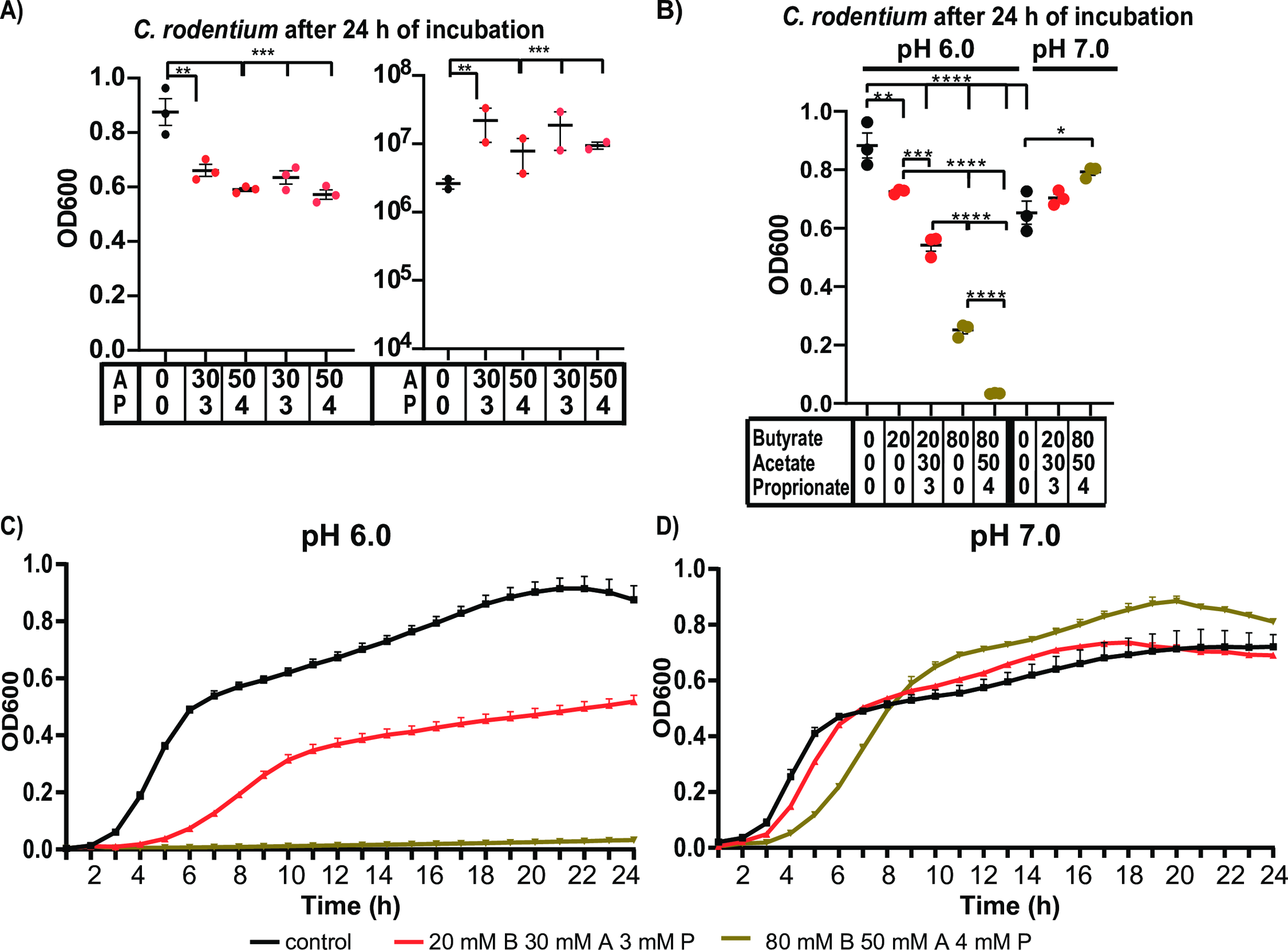

Supplement: S6 Fig — (A) C. rodentium growth displayed as optical density (OD) after 24 hours at pH 6.0 in BHI medium supplemented with different concentrations of acetate and propionate or without any SCFA added. (B) C. rodentium growth displayed as optical density (OD) after 24 hours at pH 6.0 (left) or pH (7.0) in BHI medium supplemented with different concentrations of acetate, butyrate and propionate or without any SCFA added. One data point represents mean value of three replicates. Values out of three independent experiments are displayed. P values indicated represent a one-way ANOVA between groups with **p<0.01, ***p<0.001, ****p<0.0001. (C-D) C. rodentium growth displayed as optical density over time at pH 6.0 (left) or pH (7.0) in BHI medium supplemented with different concentrations of acetate, butyrate and propionate or without any SCFA added. OD was measured every 60 min. Mean values ± SEM out of three independent experiments are displayed. (TIF) [file ppat.1008448.s006.tif]

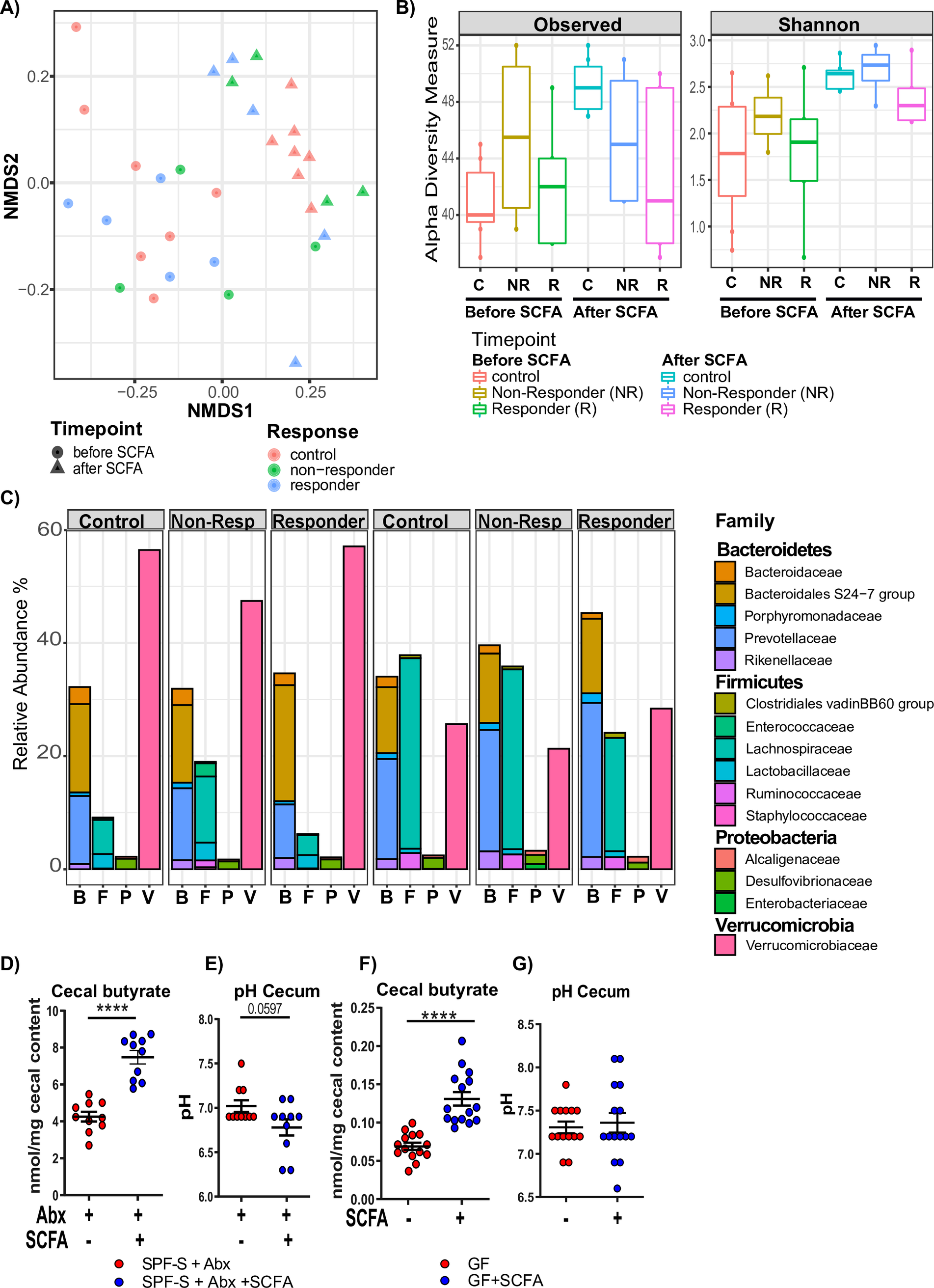

Supplement: S7 Fig — (A) Fecal bacterial microbiota composition of butyrate supplemented animals were evaluated using 16S rRNA gene sequencing regarding to their response against C. rodentium infection. β-diversity was analyzed using Bray-Curtis dissimilarity matrix and non-metric multidimensional scaling (NMDS). (B) α-diversity before and after SCFA supplementation in different groups of SPF-S mice was determined using Chao1 and Shannon index p>0.05. (C) Average microbiome level from different SPF-S mice on family level. (D-E) Cecal butyrate level and cecal pH of C57BL6/N germ-free mice supplemented with 150 mM in the drinking water at 3 days p.i. (F-G) Cecal butyrate level and cecal pH of antibiotic treated SPF-S mice supplemented with 150 mM in the drinking water at 3 days p.i. Mean and SEM of two independent experiments with n = 5–9 mice per group. P values indicated represent a nonparametric Kruskal-Wallis test *p<0.05, **p<0.01, ***p<0.001, ****p<0.0001 (TIF) [file ppat.1008448.s007.tif]
