# Supplementary material for: Variations in microbiota composition of laboratory mice influence Citrobacter rodentium infection via variable short-chain fatty acid production
Source: PLoS Pathog. 2020 Mar 24;16(3):e1008448. doi: 10.1371/journal.ppat.1008448 (PMC7141690; doi:10.1371/journal.ppat.1008448)
Supplement: Attachment — Submitted filename: Reviewers comments_Rebuttal Letter_v2_2.docx [file ppat.1008448.s010.docx]

**Responses to reviewers’ comments**:

Dear colleagues,

we very much appreciate the constructive criticism of our manuscript. In the following we provide detailed responses to the reviewers’ comments as stated in “**Part II – Major Issues: Key Experiments Required for Acceptance”**

Reviewer #1:

1.) *In Fig. 7C, the authors supplemented susceptible SPF-1 mice with a SCFA mixture consisting of butyrate, acetate and propionate in the drinking water, which correlated with lower CFU/g of C. rodentium in Fig. 7D. The authors point out that in five mice that achieved the “threshold” of 50 nmol/mg butyrate in the cecum, and lower CFU/g in the cecum (Fig. 7G), which recapitulated resistant SPF-2 mice. The authors conclude that, “…higher abundance and diversity of SCFA-producing bacteria in the cecum of SPF-2 mice lead to elevated butyrate levels and reduced pH value in the lumen of the cecum, thereby efficiently reducing growth of C. rodentium…” However, since the experiments in Fig. 7 were performed in the presence of conventional mice with a complex microbiota, it’s difficult to draw conclusions about the effects of SCFAs in these mice with such pronounced microbiota differences.*

*In Fig. 7G, the authors show that 5/19 mice achieved the “threshold” of 50 nmol/mg butyrate and lower CFU/g of C. rodentium in the cecum. However, it’s unknown whether these five mice were microbiota “outliers” with native higher levels of SCFAs independent of oral SCFA supplementation. Did the authors analyze the microbiota of these mice to examine whether the harbored higher levels of butyrate-producing microbiota or other inhibitory microbiota such as Enterobacteriaceae? Although the former observation would support their conclusions, it’s necessary to repeat these experiments in Germ-Free or antibiotic-treated mice. The authors should repeat the experiments in Fig. 7 in germ-free (or antibiotic treated mice) and show that SCFA supplementation alone is sufficient to reduce C. rodentium colonization to support their conclusion that SCFAs are responsible for these effects. The authors could also give germ-free mice butyrate-producing Firmicutes and measure SCFA, pH, and CFU/g C. rodentium in these mice.*

**Author’s response:** We have addressed the reviewer’s questions in multiple ways:

1) In response to the Reviewers questions we have analyzed the microbiota of SCFA-treated SPF1 mice used in Fig. 7 (now referred to as SPF-S mice throughout the whole manuscript and in this rebuttal letter) before and after SCFA supplementation to investigate potential differences between mice that showed a protected or susceptible phenotype. This analysis did not reveal any significant differences (see new Figure S7). Of note, SPF-S did not harbor any Enterobacteriaceae as shown in new Figure S4 (see line273-293) and new Figure S7 except *C. rodentium* after infection, thus, different levels of Enterobacteriaceae are unlikely to explain the differences.
2) To formally exclude an influence of low abundance facultative anaerobic bacteria, we first cultured and then transferred facultative anaerobic bacteria from SPF-R mice, which contained also other Enterobacteriaceae such as *C. amalonaticus*, *K. oxytoca* and *E. coli,* into SPF-S mice and assessed susceptibility to *C. rodentium*. In line with the other analysis, no direct effect of facultative anaerobic bacteria on *C. rodentium* colonization was detectable (see new Figure S4).
3) To test whether SCFA have direct activity against *C. rodentium in vivo*, we treated as suggested by the reviewer germfree and antibiotic treated mice with a mixture SCFA. SCFA supplementation of GF and antibiotic-treated SPF-S mice significantly reduced CFUs of *C. rodentium* in the cecum and colon, highlighting that SCFA alone, without the presence of commensal microbes, are able to significantly reduce the amounts of *C. rodentium* in vivo (see revised Figure 7) (see line379-388). Of note, mice devoid of complex microbiota and GF mice have low levels of butyrate, which could not be recovered by supplementation of SCFA in the drinking water to the same levels found in SPF mice. Therefore, we conclude that SCFA alone have an inhibitory effect on *C. rodentium* *in vivo*, but that this inhibitory effect is smaller in the absence of competing bacteria and is dependent on the concentrations and the pH value found in the lumen of the gut. We have now included a detailed discussion of these results in the “discussion” section of the manuscript.

Reviewer #2: Decreases in butyrate levels have been linked to increased epithelial oxygenation (Colgan group, Kelly et al., 2015 Cell Host and Microbe), and increased oxygenation enhances mucosal Citrobacter colonization (Baumler group, Lopez et al., 2016 Science). As it stands, the mechanism proposed by authors is plausible, but this alternative hypothesis cannot be formally excluded and could provide an alternative explanation of all the data. Additional experimentation is recommended to discern between these two possibilities.

**Author’s response:** We have addressed the reviewer’s suggestions in multiple ways:

1) Since the observed phenotype is pronounced in the lumen and also visible in isolated cecal content (see Figure 3), we focused on luminal effects rather than mucosal changes. In response to the Reviewers suggestion we have performed experiments to quantify HIF-1a protein expression in the tissue as marker of oxygen availability by western blotting (see new Figure S4. Notably, we did not observed remarkable differences in the expression levels of HIF1a at steady state.
2) In addition, we transfer different facultative anaerobic bacteria from SPF-R mice, which are able to consume the potentially higher amounts of oxygen in the SPF-S mice before *C. rodentium* infection (see new Figure S4). We did not detect an effect of colonization with facultative anaerobes on *C. rodentium* CFUs after 3 days p.i.

Taken together, the transfer experiments of facultative anaerobic bacteria and HIF1a western blots do not support the alternative hypothesis that increased epithelial oxygenation is the cause for the observed phenotype.

In the following we provide detailed responses to the reviewers’ comments as stated in “**Part III – Minor Issues: Editorial and Data Presentation Modifications”**

Reviewer #1:

1.) Since SCFA’s supplemented orally are readily absorbed in the small intestine, this may explain the significantly lower butyrate concentrations in Fig. 7E and CFU/g of SPF-1 + SCFA mice in Fig. 7D, relative to SPF-2 mice. Oral treatment of mice with tributyrin has been shown to increase concentrations of butyrate in the large intestine. The authors could give mice (and germ-free or antibiotic treated) tributyrin instead of free SCFA to see if this could restore butyrate levels and reduce colonization in mice.

Author’s response: We appreciate the reviewer’s suggestion of using tributyrin instead of SCFA to reduce colonization of *C. rodentium in vivo*. While we are convinced that tributyrin supplementation could be an interesting avenue to follow-up, we decided against utilizing in the current manuscript for several reasons:

- The reviewer suggested to provide SCFA to germfree and antibiotic-treated mice to show the direct effects of SCFA in vivo. To perform the experiments in a comparable manner to the experiments in Figure 7, we decided to use SCFA and not tributyrin for these experiments.
- In order to identify the optimal dosing of tributyrin resulting in physiological cecal butyrate concentration a specific dose-finding experiment is required. In order to perform these experiments, we have to apply for a separate animal license as our current license does not cover this kind of intervention. The application for such license currently takes up to 4 months in Germany, which would have immensely delayed the submission of the revised manuscript.

2.) It would be helpful for the reader if the authors changed the names of the different mice from SPF-1 and SPF-2 to SPF-S and SPF-R, respectively, to make it easier to follow with mice are resistant and which are susceptible.

Author’s response: In response to the Reviewers suggestion, we have changed the name of the different mouse lines SPF-1 and SPF-2 to SPF-S and SPF-R mice, respectively, in the whole manuscript and in this rebuttal letter.

Reviewer #2: Data presentation is very clear and concise.

Author’s response: Data presentation of the revised manuscript is in line with the original manuscript.
